# POLE2 knockdown reduce tumorigenesis in esophageal squamous cells

**DOI:** 10.1186/s12935-020-01477-4

**Published:** 2020-08-11

**Authors:** Yongjun Zhu, Gang Chen, Yang Song, Zhiming Chen, Xiaofeng Chen

**Affiliations:** Department of Cardiothoracic Surgery, Huashan Hospital, Fudan University, No. 12, Mid, Wulumuqi Rd, Shanghai, China

**Keywords:** ESCC, POLE2, Cell proliferation, Cell apoptosis, Cell migration

## Abstract

**Background:**

Esophageal squamous cell carcinoma (ESCC) is one of the most frequent malignant tumors originated from digestive system around the world and the treatment was limited by the unclear mechanism. DNA polymerase epsilon 2, accessory subunit (POLE2) is involved in DNA replication, repair, and cell cycle control, whose association with ESCC is still not clear.

**Methods:**

In this study, the expression level of POLE2 in ESCC tissues was detected by IHC. The POLE2 knockdown cell line was constructed, identified by qPCR and western blot and used for detecting cellular functions and constructing xenotransplantation mice model. MTT Assay, colony formation assay, flow cytometry, wound-healing assay and Transwell assay were used to detected cell proliferation, apoptosis and migration.

**Results:**

We firstly identified that the expression of POLE2 was overexpressed in ESCC. Moreover, the high expression of POLE2 can predict the tumor deterioration and poor prognosis of ESCC patients. Additionally, downregulation of POLE2 was involved in ESCC progression by promoting proliferation, migration, and inhibiting apoptosis in vitro. In vivo studies proved that POLE2 was positively correlated with ESCC tumor formation, which was consistent with the results in vitro. We also illuminated that POLE2 knockdown upregulated pro-apoptotic proteins (Bax, Caspase3, CD40L, FasL, IGFBP-5 and P21) and downregulated anti-apoptotic proteins (CLAP-2, IGF-I and sTNF-R2). In addition, POLE2 was involved in ESCC via targeting PI3K/Akt, Cyclin D1 signaling pathway.

**Conclusions:**

Therefore, POLE2 was proved to be involved in the development of ESCC, which may be a potential therapeutic target and bring new breakthroughs in the treatment of ESCC.

## Introduction

Esophageal cancer (EC) is one of the most aggressive gastrointestinal cancers in the world [1]. Esophageal squamous cell carcinoma (ESCC) is one of the main subtypes of EC. Although the incidence of ESCC has shown a downward trend in recent years, the morbidity and mortality of the disease remain high worldwide [2]. At present, the comprehensive treatment ESCC consists of surgery and definitive chemoradiotherapy, supplemented with cisplatin and 5-fluorouracil (5-FU) (CF) [3]. Moreover, it was reported that agents inhibiting erb-b2 receptor tyrosine kinase 2 (ERBB2 or HER2), and vascular endothelial growth factor (VEGF), such as Trastuzumab, Ramucirumab and Lapatinib, could improve survival of ESCC patients [4]. Unfortunately, the treatments of ESCC could only relieve symptoms, but the prognosis is still poor, especially with a 5-year survival rate of less than 40% [5, 6]. Thus, in attempted to improve outcomes of ESCC, novel therapeutic or preventive strategies are urgently needed.

The eukaryotic DNA polymerase epsilon was first isolated from Saccharomyces in 1970, and belongs to the DNA polymerase B family, which contains four subunits. The largest subunit is POLE (subunit A), the second largest subunit is POLE2 (subunit B) with a molecular weight of 59 kDa, respectively [7]. POLE2 is involved in cellular functions such as DNA replication, repair, and cell cycle control [8], as well as in the array-based proliferative signatures [9]. On the other hand, POLE2 was previously reported to be abnormally expressed in breast, colorectal, cervical and bladder cancer [10–13]. For example, Zhou et al. reported that genomic changes of the 55 kDa Subunit of DNA Polymerase ε in human breast cancer [10]. Daniel et al. believed that POLE2 mutation will affect colorectal cancer and make contribution to the heritable risk of colorectal cancer [11]. Yet to date, the role of POLE2 played in ESCC is still not clear. Thus, further study is needed to evaluate and identify the role of POLE2 in ESCC development and progression.

The results of this study indicated that POLE2 was significantly upregulated in ESCC. Knockdown of POLE2 could suppress ESCC development through regulating cell proliferation, apoptosis and migration. Therefore, this study suggested that POLE2 was involved in the development of ESCC and may be a potential therapeutic target, providing a new therapeutic strategy for preventing or delaying the progression of ESCC.

## Materials and methods

### Immunohistochemical staining

The 105 pairs of ESCC tissues and matched non-cancer normal tissues were purchased from Shanghai Outdo Biotech Company, with detailed pathological data. Collection of samples with informed consent from patients and the study were approved by the Institutional Animal Care and Use Committee of Fudan University.

The formalin fixed tissue samples were first hydrated with xylene for 15 min and then with 100% ethanol for 10 min. After repairing and blocking of the citrate antigen, the sample and POLE2 antibody (1: 200, BIOSS, USA, Cat. # BS-14356R) were incubated overnight in an incubator at 4 °C. After elution with PBS for 5 times, secondary antibody IgG (1: 400, Abcam, USA, Cat. # AB6721) was added, incubated at room temperature for 30 min, and washed with PBS for 3 times. Tissue slices were first stained with DAB, and then with hematoxylin. Finally, images were collected with a photomicroscope and analyzed according to the German immune response score [14]. The expression level of POLE2 was defined by the total score of IHC staining. Notably, the high or low expression level was distinguished by the median of total score of IHC.

### Cell culture

ESCC cell lines Eca-109 and TE-1 were obtained from the Cell Bank of the Chinese Academy of Sciences (Shanghai, China). They were grown in 6-well plates with 5% CO_2_ in the wetted air, using DMEM supplemented with 10% FBS (Hyclone, USA, Cat. # SV30087) at 37 °C. The medium was changed every 72 h.

### Target gene RNA interferes with the preparation of lentiviral vector

The target sequence of RNA interference (5′-CGATTGTTCTTGGAATGATA-3′) was designed using POLE2 gene as template to build target gene RNA interference lentivirus vector. The 20 µL reaction system was prepared according to the T4 DNA Ligase, and the double-stranded DNA oligo was connected to the linearized carrier (100 ng/L). After the ligation products were transformed into TOP10 *E. coli* competent cells (100 µL, TIANGEN, Beijing, China, Cat. # CB104-03), 500 µL LB liquid medium without antibiotics was added, and it was conducted in a shaking culture at 37 °C for 1 h. 150 µL bacterial solution was evenly applied to LB solid medium containing Amp and cultured overnight in 37 °C incubator. A 20 µL PCR reaction system was prepared, and a single colony was picked up as a template. The reaction conditions were: 94 °C for 3 min; 94 °C for 30 s, 55 °C for 30 s, 72 °C for 30 s, 22 cycles; 72 °C for 5 min. The bacteria with correct sequencing was selected. Subsequently, according to the kit instructions, plasmids was extracted (TIANGEN, Beijing, China, Cat. # DP117). Lentivirus expressing shPOLE2 or shCtrl were constructed by Bioscienceres Co. Ltd (Shanghai, China). The efficiency of the transfection of cells by lentivirus were evaluated by the detection of fluorescence intensity in cells (green fluorescence protein tag on lentivirus).

### qPCR

Firstly, total RNA was extracted according to Trizol instructions (Invitrogen, Carlsbad, CA, USA). Nanodrop 2000/2000C spectrophotometric were used to analysis the quality of extracted RNA and relative levels of the mRNAs. Reverse Transcription Kit (Vazyme, Nanjing, China) was used to synthesize cDNAs. The real-time reverse transcription PCR was performed by using AceQ qPCR SYBR Green Master Mix (Vazyme, Nanjing, China). GAPDH was used as a reference control. The qPCR was analyzed by 2^−∆∆CT^ method and collected data.

**Table:** 

Target name	Sequence (5′ → 3′)
POLE2 primer-F	TGAGAAGCAACCCTTGTCATC
POLE2 primer-R	TCATCAACAGACTGACTGCATTC
GAPDH primer-F	CGGATTTGGTCGTATTGGG
GAPDH primer-R	GATTTTGGAGGGATCTCGC

### Western blot analysis

The Eca-109 and TE-1 cells were collected and lysed with RIPA lysis buffer (Cell Signal Technology, Danvers, MA, USA) according to the instructions. The proteins detection with BCA protein assay Kit (HyClone-Pierce, Waltham, MA, USA, Cat. # 23225). Each protein was transferred to polyvinylidene fluoride (PVDF) membrane in 20 µg quantities and incubated with 5% BSA in Tris-buffered saline containing 0.5% Tween 20 for 60 min, and then incubated overnight at 4 °C on a rocker with the following primary antibodies: POLE2, Akt, p-Akt, MAPK9, Cyclin D1, PIK3CA and GAPDH antibody. Following washing three times with TBST for 5 min, blots were then incubated with horseradish peroxidase (HRP) conjugated goat anti-rabbit IgG polyclonal secondary antibody (Goat Anti-Rabbit, 1:3000) (Beyotime, Beijing, China, # A0208) at room temperature for 1 h. Immunoreactive blots were viewed by ECL and plus TM western blotting system kit (Amersham, Chalfont, UK, Cat. # RPN2232) and band intensity was quantified using Image J software.

**Table:** 

Name of antibody	Protein size (kDa)	Diluted multiples	Source of primary antibody	Company	Number
POLE2	59	1:1000	Rabbit	Abcam	AB180214
Akt	60	1:1000	Rabbit	CST	4685
p-Akt	60	1:1000	Rabbit	Bioss	BS5193R
MAPK9	48	1:1000	Rabbit	Abcam	AB76125
Cyclin D1	36	1:2000	Rabbit	CST	2978
PIK3CA	110	1:1000	Rabbit	Abcam	AB40776
GAPDH	37	1:3000	Rabbit	Bioworld	AP0063

### MTT assay

The Eca-109 and TE-1 cells were cultured to logarithmic growth phase and digested with trypsin, then cells (2000 cells/well) were re-suspended and inoculated to 96-well plates (100 µL/well) (Corning, Corning, NT, USA, Cat. # 3599) overnight. 20 µL MTT (5 mg/mL) (3-(4, 5-dimethylthiazol-2-yl)-2, 5-diphenyl tetrazolium bromide) (Genview, Beijing, China; Cat. # JT343) was added to each well within 4 h prior to culture termination. 100 µL DMSO was added to lyse Formazan crystal. OD value was detected after oscillating for 5 min with the enzyme-connected immunodetector 490/570 nm. The cell viability ratio was calculated by the following formula: cell viability (%) = OD (treated)/OD (control) × 100%.

### Colony formation assay

Lentivirus-mediated Eca-109 and TE-1 cells were digested with trypsin, then suspended and counted, and inoculated in 6-well plates at 400–1000 cells per well. Cells were incubated for 14 days to form colonies and the medium was changed every 3 days. Then the cells were washed with PBS, fixed with paraformaldehyde for 1 h, stained with Giemsa for 20 min and washed three times by ddH_2_O. Finally, photographed with digital camera and counted with fluorescence microscopy (Micro Publisher 3.3RTV; Olympus, Tokyo, Japan).

### Apoptotic assay

Lentiviral Eca-109 and TE-1 cells transfected for 5 days were inoculated in culture dishes, digested with trypsin, suspended, and stained with Annexin V-APC (10 µL) in the dark for 15 min. Cell apoptosis rates were assessed using FACScan (Millipore, Darmstadt, Germany, USA). Meanwhile, the green fluorescence of GFP labeled on lentivirus was detected, as shown in the Y-axis. The cells not transfected with lentivirus were excluded. Therefore, the rate of apoptosis was accurately determined.

### Wound-healing assay

Eca-109 and TE-1 Cells (3 × 10^4^/well) were seeded into 6-well dishes and grew until 90% confluence. A line wound was made by a pipette tip across the cell layer. The cells were washed with PBS and cultured in an incubator with 5% CO_2_ at 37 °C. Photograph were captured by fluorescence micrograph at the time point (0 h, 24 h and 48 h; 0 h, 3 h and 6 h). Cell mobility was calculated according to the measurement.

### Transwell assay

The required number of Chambers (24-well, 8-mm pore) (NY, USA) were placed in an empty 24-well plate, 100 μL serum free medium was added to the chamber, and the incubator was placed at 37 °C for 1 ~ 2 h. Trypsin digested the cells of each group at logarithmic growth phase, then the cell was suspended with low serum medium and counted. The cell suspension 100 μL (total 5 × 10^5^ cells) was loaded into the upper chamber of the Transwell and the lower chamber of Transwell was filled with 500 μL culture mediums containing 30% FBS for incubation 48 h at 37 °C. The non-invading cells on the upper chamber were removed with a cotton swab, while the cells adhering to the membrane were fixed in 4% paraformaldehyde (precooled with ice) for 30 min and stained with 0.1% crystal violet for 20 min at room temperature. After PBS washing, 5 fields of each well were randomly selected under the microscope, and images were taken to count the cells.

### Human apoptosis antibody array

The intracellular cell signaling pathway was examined using the Human Apoptosis Antibody Array Kit (Cat. # AB134001). In short, the cells were collected 3 days after lentivirus transfection, washed with PBS, and then lysed with lysis buffer at 2 ~ 8 °C for 30 min, which was gently shaken. The total extracted protein was diluted with the Array Diluent Buffer Kit (0.5 mg/mL). Each array of antibody membrane was blocked with blocking buffer for 30 min at room temperature, which incubated at 4 °C and gently shaken overnight. Membranes were incubated with 1× Biotin-conjugated Anti-Cytokines overnight at 4 °C with gentle shaking. HRP linked Streptavidin was added to the membranes. Protein was visualized using ChemiDoc XRS chemiluminescence detection (HyClone-Pierce, Cat. # 23225; Amersham, RPN2232) and imaging system. The density of the spots was quantitated using Quantity One software and normalized to the *α*–tubulin levels.

### Animal xenograft model

The animal experiment was approved and performed according to the guidelines of the Institutional Animal Care and Use Committee of Fudan University. BALB/c female nude mice (4 weeks old) were purchased from Shanghai Jiesijie Experimental Animals Co., Ltd (Shanghai, China). The Eca-109 cells (4 × 10^6^ per mouse) transfected by shPOLE2 or shCtrl were subcutaneously injected into BALB/c female nude mice. Data (weight and volume of the tumor) were collected 15 days after the injection of Eca-109 cells, followed by weekly measurements up to 25 days. Tumor burden was assessed weekly by bioluminescence imaging and analyzed by IVIS Spectrum Imaging System (emission wavelength, 510 nm). 10 min before in vivo imaging, anesthesia was performed by inhaling with 3% isoflurane. After 25 days mice were sacrificed through injection of pentobarbital sodium, and the tumors were removed for taking photos and weighting.

### Ki67 staining

Mice tumor tissues were fixed in 10% formalin and then immersed in xylene and ethanol to dewax and rehydrate. After the tissues were repaired and blocked with the citrate antigen, incubated with antibody Ki67 (1: 200, Abcam, USA, Cat. # AB16667) overnight at 4 °C. After 5 times of elution with PBS, added secondary antibody IgG (1: 400, Abcam, USA, Cat. # AB6721) and incubated at room temperature for 30 min, then washed with PBS for 3 times. Tissue slices were first stained with DAB, and then with hematoxylin. Images were collected with a photomicroscope and analyzed.

### Statistical Analysis

The data are expressed as mean ± SD (n ≥ 3) and analyzed using GraphPad Prism 6 software (GraphPad Software Inc., San Diego, CA, USA). T-test were used to compare the difference. *P* value less than 0.05 were considered statistically significant. All experiments were performed in triplicate and data were presented as mean ± SDs.

## Results

### Upregulation of POLE2 in ESCC tissues

**Fig. 1:**
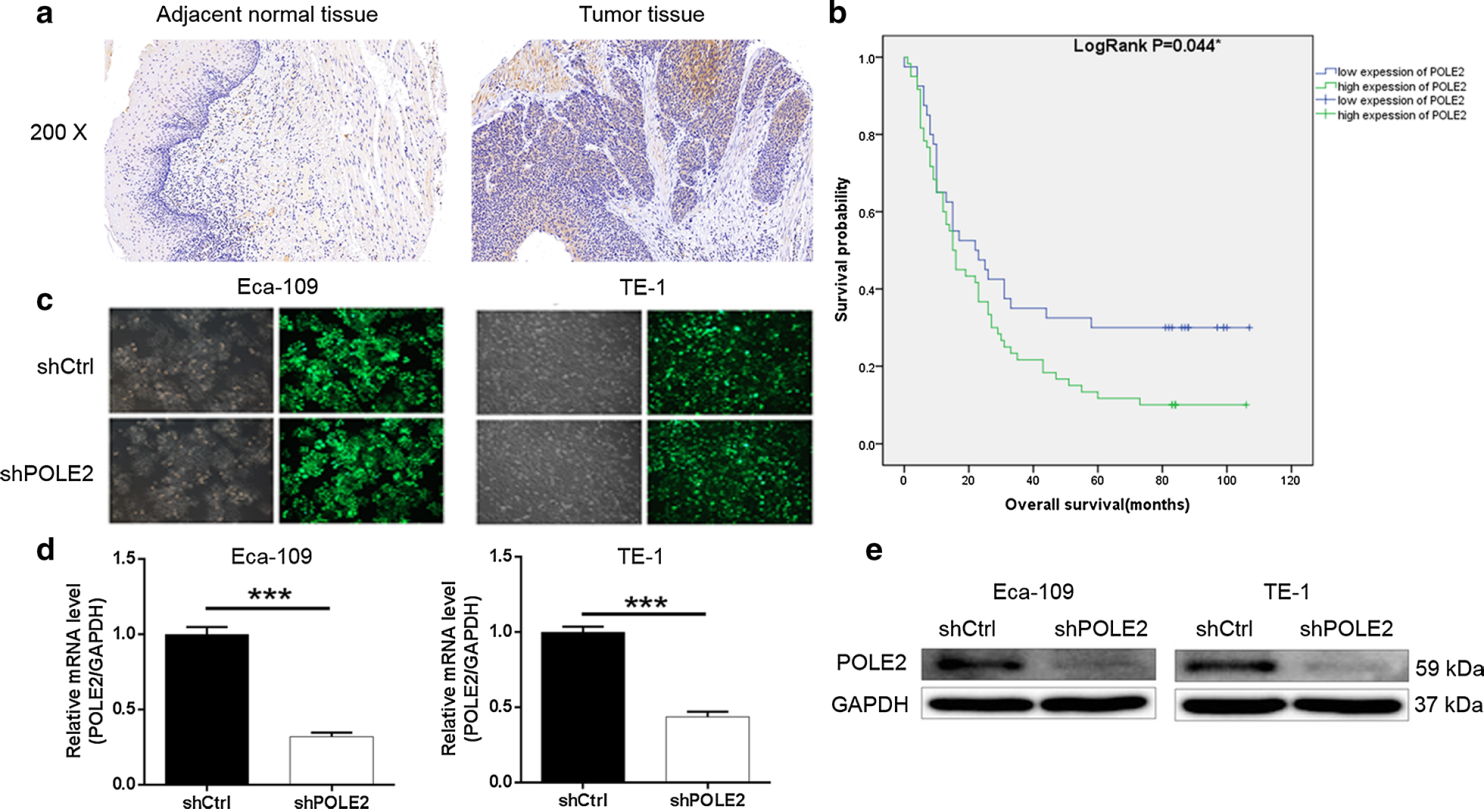
POLE2 is highly expressed in ESCC tissues and the construction of POLE2 knockdown cell model. **a** Expression levels of POLE2 in ESCC tumor tissues and normal skin tissues were detected by IHC staining. **b** POLE2 expression and overall survival of ESCC by Kaplan–Meier survival analysis. **c** Transfection efficiency for Eca-109 and TE-1 cells was evaluated by expression of green fluorescent protein 72 h post-infection. **d**, **e** The specificity and validity of the lentivirus-mediated shRNA knockdown of POLE2 expression was verified by qPCR (**d**) and western blot analysis (**e**). The data were presented as the mean ± SD (n = 3), *P < 0.05, **P < 0.01, ***P < 0.001

Firstly, the expression of POLE2 in ESCC tissues and normal tissues were detected by immunohistochemical staining (Fig. 1a). As shown in Table 1, generally and significantly higher expression levels of POLE2 were observed in tumor tissues compared with normal tissues (*P *< 0.001), indicating the potential involvement of POLE2 in the development and progression of ESCC. Subsequently, based on the Mann–Whitney U analysis, we clarified that high POLE2 expression was positively correlated with gender (male), advanced tumor stage and high risk of lymphatic metastasis by the Mann–Whitney U analysis (Table 2). These results were further verified by Spearman grade correlation analysis (Table 3), indicating the upregulation of POLE2 with the deepening of tumor malignancy. More importantly, according to the Kaplan–Meier survival (Fig. 1b) we found that POLE2 expression was negatively correlated with overall survival of ESCC. To sum up, it can be concluded that expression of POLE2 may be a predictor of poor prognosis for patients with ESCC.

**Table 1:** Expression patterns in esophagus cancer tissues and para-carcinoma tissues revealed in immunohistochemistry analysis

POLE2 expression	Tumor tissue		Para-carcinoma tissue		*P* value
	Cases	Percentage	Cases	Percentage	
Low	41	40.6	64	100	0.000***
High	60	59.4	0	–

**Table 2:** Relationship between POLE2 expression and tumor characteristics in patients with esophagus cancer

Features	No. of patients	POLE2 expression			*P* value
		Low		High	
All patients	101	41		60	
Age (years)					0.601
≤ 65	51	22		29	
> 65	50	19		31	
Gender					0.040*
Male	75	26		49	
Female	26	15		11	
Lymph node positive					0.100
≤ 1	52	24		28	
> 1	38	11		27	
Tumor size (cm)					0.757
< 5	41	16		25	
≥ 5	42	15		27	
Grade					0.635
I	8	3		5	
II	66	26		40	
III	27	12		15	
AJCC stage					0.002**
1	4		2	2	
2	42		25	17	
3	50		13	37	
T infiltrate					0.623
T1	4		2	2	
T2	11		5	6	
T3	80		33	47	
T4	3		1	2	
Lymphatic metastasis (N)					0.006**
N0	45		26	19	
N1	30		7	23	
N2	19		6	13	
N3	4		1	3

**Table 3:** Relationship between POLE2 expression and tumor characteristics in patients with esophagus cancer

POLE2		*P* value
AJCC stage	Pearson correlation	0.319
	Significance (double tailed)	0.002**
	N	96
Lymphatic metastasis (N)	Pearson correlation	0.277
	Significance (double tailed)	0.006**
	N	98
Gender	Pearson correlation	− 0.205
	Significance (double tailed)	0.040*
	N	101

### Construction of POLE2 knockdown in cell models

ESCC cell line Eca-109 and TE-1 were chosen as cell models for subsequent experiments. The cells were transfected with shPOLE2 for silencing POLE2, while that transfected with shCtrl were used as negative control. The transfection efficiencies in Eca-109 and TE-1 cells were verified to be above 80% by fluorometric analysis (Fig. 1c). Compared with the shCtrl groups, the results of qPCR displayed that the knockdown efficiencies of POLE2 in Eca-109 and TE-1 cells were 67.9% and 56.2%, respectively (Fig. 1d). Similar trend was also observed in western blot (Fig. 1e). Therefore, our data suggested that POLE2 knockdown cell models were successfully constructed.

### Knockdown of POLE2 inhibited ESCC cell proliferation and colony formation

**Fig. 2:**
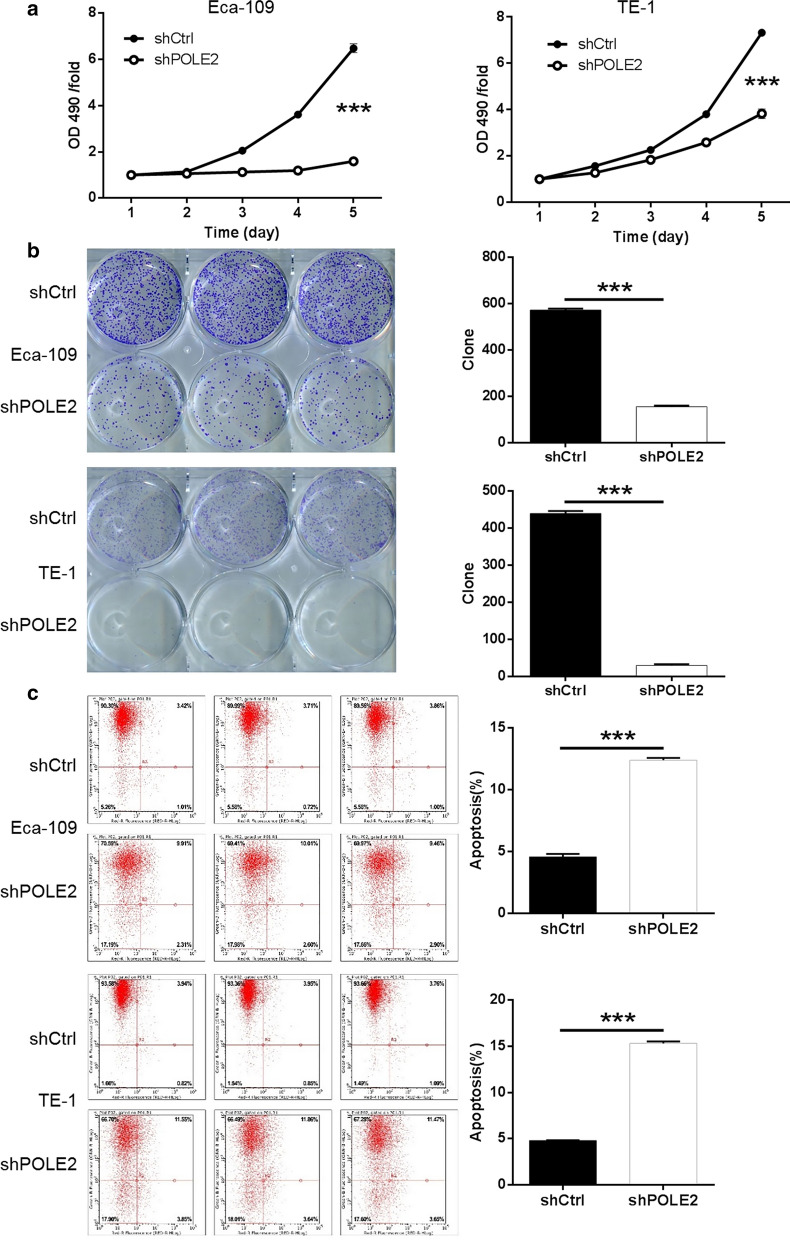
Knockdown of POLE2 inhibits cell proliferation, promotes apoptosis in ESCC cells. **a** Cell proliferation of Eca-109 and TE-1 cells with or without knockdown of POLE2 was evaluated by MTT assay. **b** Colony formation was evaluated for Eca-109 and TE-1 cells with or without POLE2 knockdown. **c** Flow cytometry analysis based on Annexin V-APC staining was utilized to detect the percentage of early apoptotic cell for Eca-109 and TE-1 cells. The X axis indicated the cell apoptosis while the Y axis indicated the green fluorescence detected from the GFP tagged on lentivirus (shPOLE2 or shCtrl). The data were expressed as mean ± SD (n = 3), *P < 0.05, **P < 0.01, ***P < 0.001

To further investigate the role of POLE2 in the development of ESCC, MTT assay and colony formation assay was accomplished. As far as the value of OD490/fold is concerned, the values of Eca-109 and TE-1 in shPOLE2 group were consistently lower than those in shCtrl, group, which indicated that the down regulation of POLE2 inhibits cell viability (*P* < 0.001) (Fig. 2a). Secondly, colony formation capability is another character for malignant tumors. Giemsa staining was performed to determine the impacts of POLE2 knockdown on colony formation in ESCC cells and the cell colony numbers were quantified. As illustrated in Fig. 2b, it was visible to the naked eye that the number of cell clones in the shPOLE2 group was obviously less than that in the control group, suggesting that POLE2 knockdown inhibited colony formation ability of Eca-109 and TE-1 cells (*P* < 0.001). The results suggested that knockdown of POLE2 may resulted in suppressing cell proliferation and colony formation in ESCC.

### Knockdown of POLE2 promoted ESCC cell apoptosis

Subsequently, flow cytometry was applied to evaluate the percentage of apoptotic cells among the cells transfected with shPOLE2 or shCtrl (indicated by Y-axis: the green fluorescence from GFP on lentivirus). The average apoptosis rate of in Eca-109 and TE-1 cells increased by about 7.82% and 10.54%, respectively in the shPOLE2 group comparison to the shCtrl groups (*P* < 0.001) (Fig. 2c), indicating that the knockdown of POLE2 considerably greatly facilitated cell apoptosis in Eca-109 and TE-1 cells in comparison to the shCtrl groups. The apoptosis rates in Eca-109 and TE-1 cells increased by about 7.82% and 10.54%, respectively (*P *< 0.001) (Fig. 2c). Our results indicated that the knockdown of POLE2 notably significantly increased the apoptosis sensitivity of ESCC cells promoted the apoptosis in ESCC cells.

### Knockdown of POLE2 inhibited ESCC cell migration

**Fig. 3:**
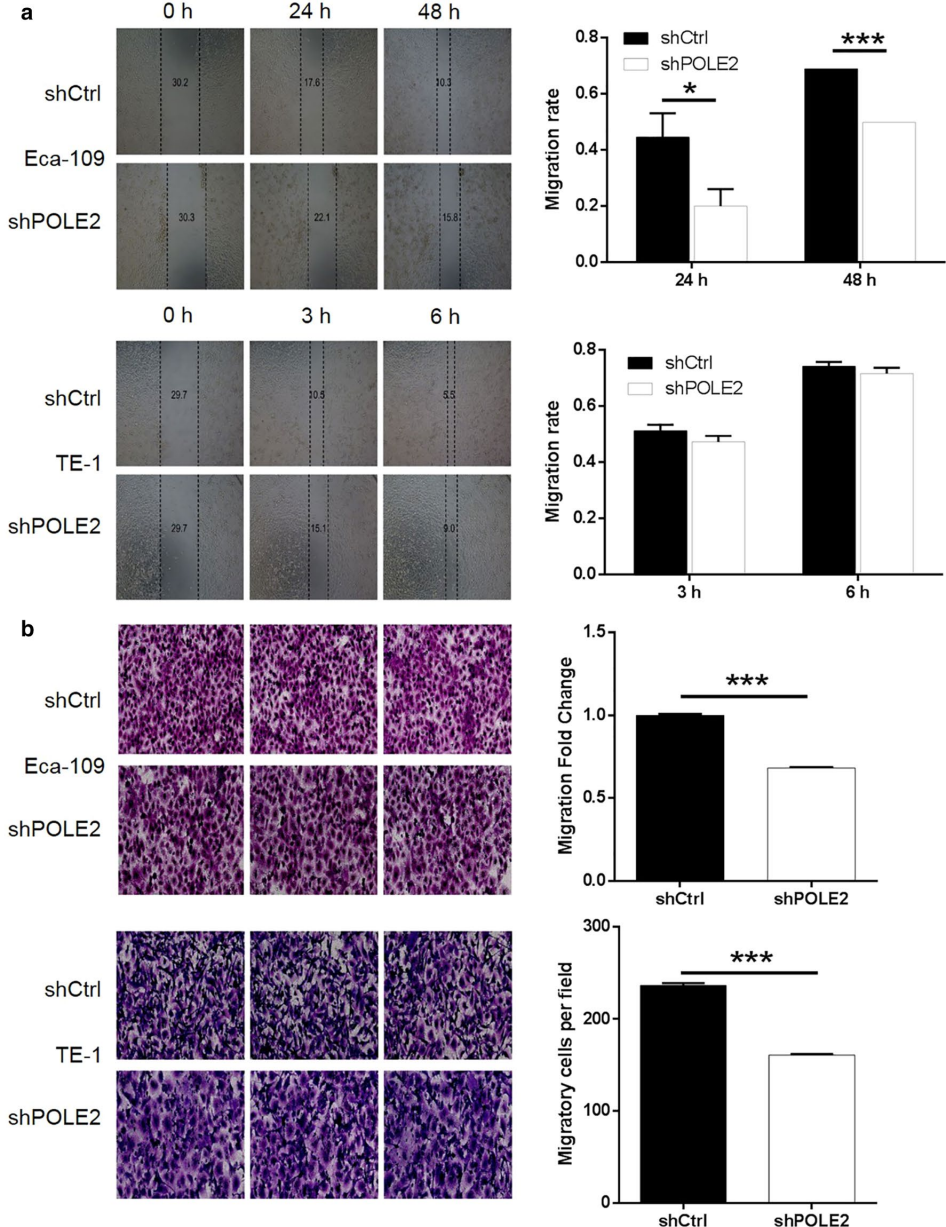
Knockdown of POLE2 inhibits cell migration in ESCC cells. **a** Cell migration of Eca-109 and TE-1 cells with or without knockdown of POLE2 was evaluated by wound-healing assay. **b** Cell migration of Eca-109 and TE-1 cells with or without knockdown of POLE2 was evaluated by Transwell assay. The data were expressed as mean ± SD (n = 3), *P < 0.05, **P < 0.01, ***P < 0.001

To evaluated the role of POLE2 in the metastasis of ESCC, cell migration ability was completed by wound-healing assay and Transwell assay. In wound-healing assay, the migration rate during 48 h of Eca-109 cells in shPOLE2 group sharply decreased by 28% than that the shCtrl group (*P* < 0.001). In contrast, the migration rate of TE-1 cells exhibited no significant alteration in shPOLE2 group compared with shCtrl group (Fig. 3a). Moreover, the results of Transwell assay presented that the number of stained cells of Eca-109 and TE-1 observed under objective lens in shPOLE2 group was remarkably less than that in shCtrl group. In addition, the migration fold change of Eca-109 and TE-1 cells in shPOLE2 group was greatly decreased over 30% (*P* < 0.001, Fig. 3b). Combined with the above two results, it can be concluded that the downregulation of POLE2 inhibits the migration ability of ESCC cells.

### Human apoptosis antibody array analysis of POLE2 knockdown in ESCC cell

**Fig. 4:**
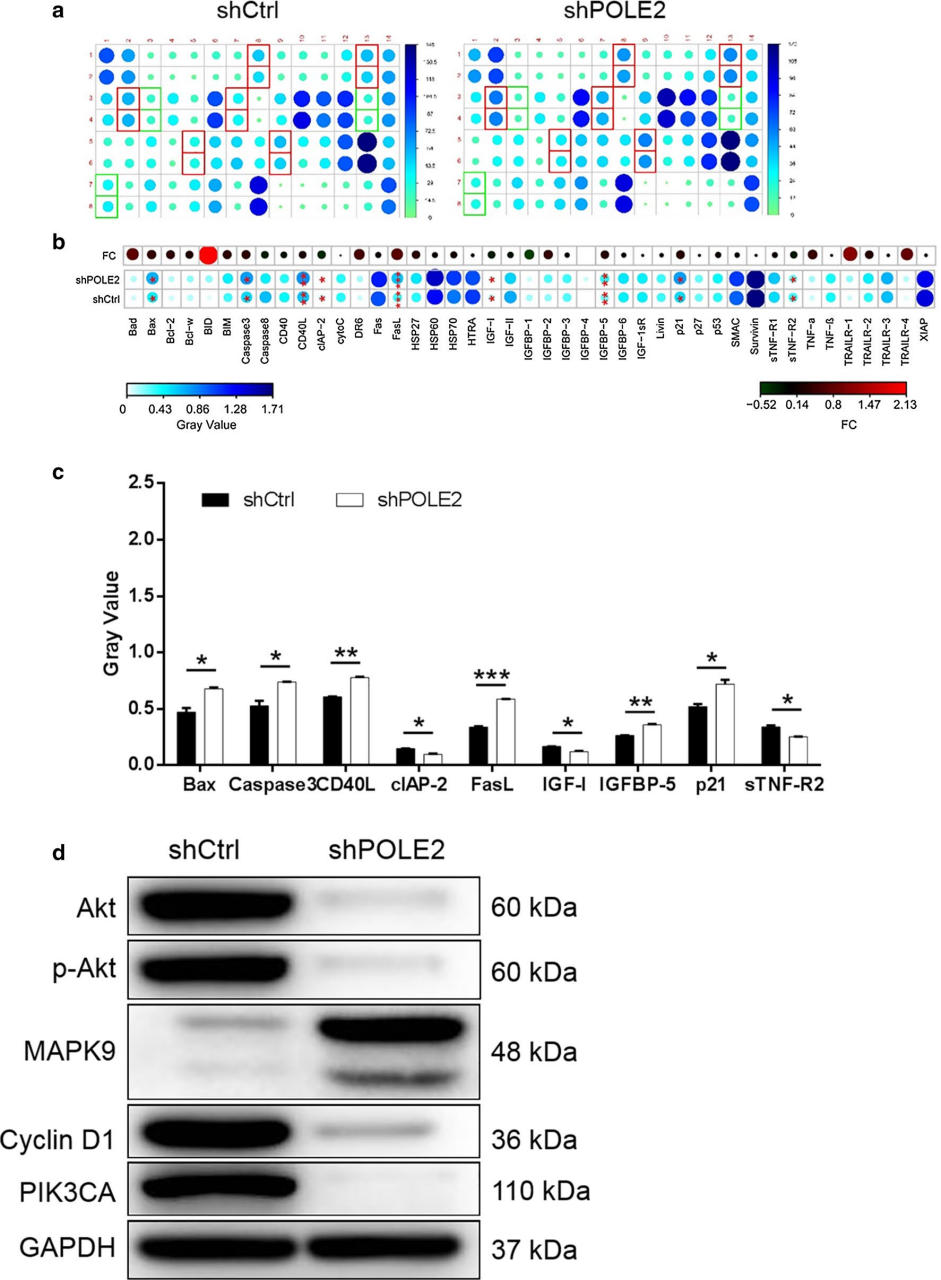
Exploration of downstream molecular mechanism of POLE2 in ESCC cells. **a** Human apoptosis antibody array analysis was performed in Eca-109 cells with or without POLE2 knockdown. **b** Differences in human apoptotic antibody array were analyzed in Eca-109 cells regardless of POLE2 knockdown. **c** Densitometric analysis was performed and the gray values of differentially expressed proteins were shown. **d** The expression of pathway was observed by western blot in Eca-109 cells. The data were expressed as mean ± SD (n = 3), *P < 0.05, **P < 0.01, ***P < 0.001

For exploring the potential mechanism of the regulation ability of POLE2 knockdown in ESCC, human apoptosis antibody array was performed to analyze the differential expression of 43 proteins in Eca-109 cells between shPOLE2 and shCtrl groups. According to Fig. 4a–c, downregulation of POLE2 in Eca-109 cells induced significant upregulation of pro-apoptotic proteins including Bax, Caspase3, CD40L, FasL, IGFBP-5, P21 and the significant downregulation of anti-apoptotic proteins including CLAP-2, IGF-I and sTNF-R2. These results were in consistent with the cellular experiments especially the cell apoptosis assay. Moreover, the detection of corresponding pathway by western blot showed that the expression levels of Akt, p-Akt, Cyclin D1 and PIK3CA in the shPOLE2 group were downregulated compared with shCtrl, while the expression of MAPK9 was upregulated (Fig. 4d).

### Knockdown of POLE2 in ESCC cell impaired tumorigenesis in vivo

The results validated that the downregulation of POLE2 could inhibit the progression of ESCC cells in vitro, but it remained to be determined whether this result can be followed in vivo. Therefore, Eca-109 cells with or without POLE2 knockdown were subcutaneously injected into nude mice to establish a mouse xenotransplantation model.

**Fig. 5:**
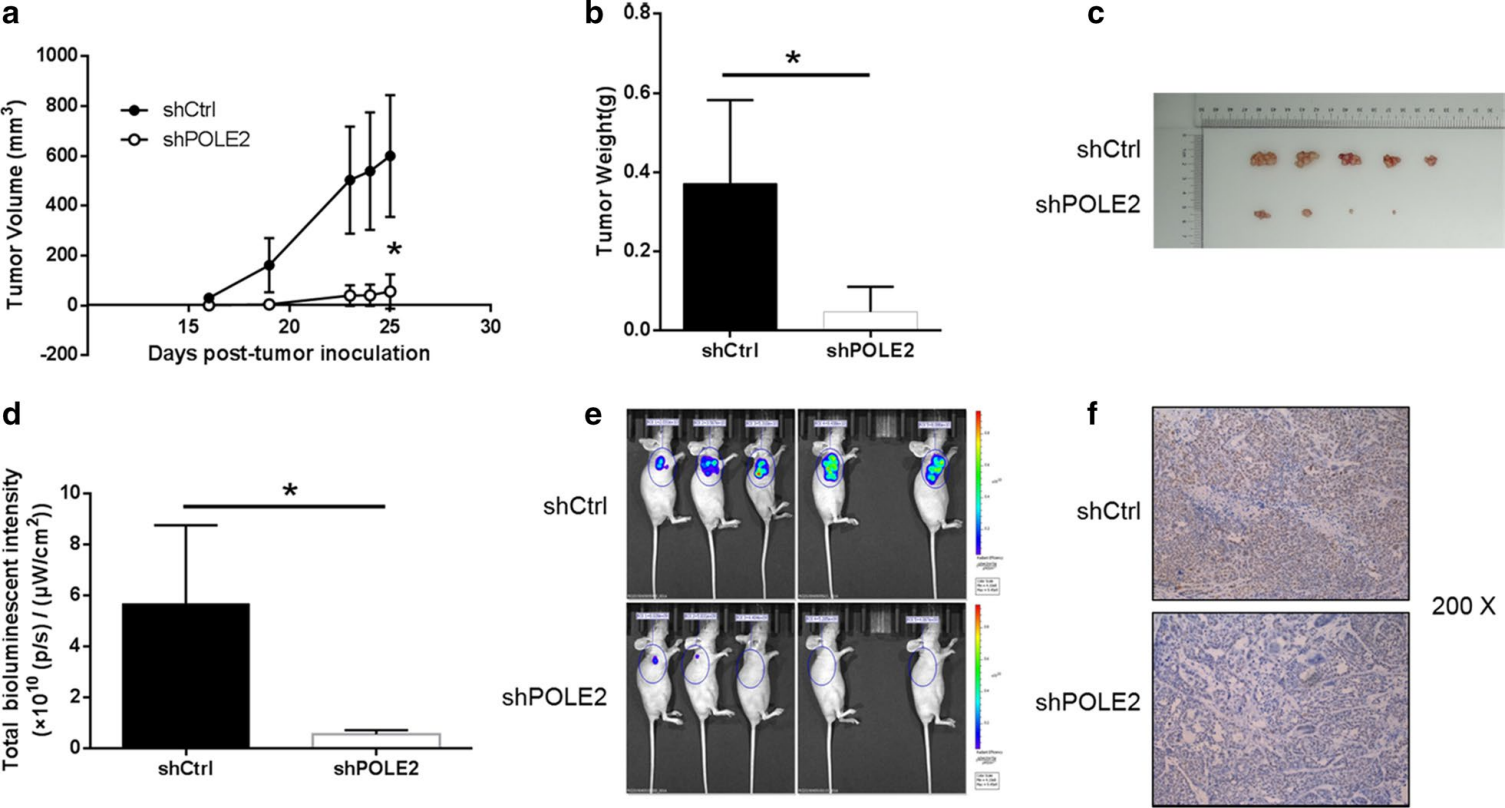
Knockdown of MEX3A inhibits tumor growth in mice xenograft models. **a** The volume of tumors in shCtrl group and shPOLE2 group was measured post-injection. **b** The average weight of tumors in shCtrl group and shPOLE2 group. **c** The picture of tumors taken from mice in shCtrl group and shPOLE2 group. **d** The total bioluminescent intensity of tumors in shCtrl group and shPOLE2 group. **e** The bioluminescence imaging of tumors in shCtrl group and shPOLE2 group. **f** The Ki67 staining of tumor tissues in shPOLE2 group and shPOLE2 group. The data were expressed as mean ± SD (n = 3), *P < 0.05, **P < 0.01, ***P < 0.001

In terms of tumor volume, continuous measurements showed that the shPOLE2 group was significantly smaller than the shCtrl group (*P* < 0.05) (Fig. 5a). Unsurprisingly, the average weight of the tumor removed from the mice in the shPOLE2 group was much smaller 324.0 ± 230 mg than that in the control group (*P* < 0.05) (Fig. 5b). As illustrated in Fig. 5c, the tumor removed from the mice more intuitively presented that the downregulation of POLE2 weakens tumor formation. In addition, the results of bioluminescence imaging showed that the fluorescence intensity of tumor in shPOLE2 group was much weaker than that in shCtrl group, indicating that POLE2 knockdown inhibited tumor formation (Fig. 5d, e). Similarly, the representative pictures of Ki67 staining also presented that the number of positive cells in the shPOLE2 group was significantly less than that in the control group (Fig. 5f). Taken together, these results suggested that downregulation of the expression of POLE2 can reduce the ability of tumor formation in mice in vivo, which was consistent with the data in vitro.

## Discussion

POLE2 is the DNA polymerase epsilon B-subunit and participates in DNA replication, repair, and cell cycle control [8, 15]. Mutations in POLE2 have been reported in patients with combined immunodeficiency, facial deformities and autoimmune deficiency [16]. In addition, abnormalities of POLE2 have also been found in human tumors, such as colorectal cancer [16, 17]. Downregulation of POLE2 expression could significantly inhibit the proliferation and colony formation and induce apoptosis of lung adenocarcinoma cell [18]. However, the role of POLE2 in ESCC is not clear.

In this study, we firstly identified that the expression of POLE2 was overexpressed in ESCC. Moreover, the high expression of POLE2 can predict the tumor deterioration and poor prognosis of ESCC patients. Additionally, downregulation of POLE2 was involved in ESCC progression by promoting proliferation, migration, and inhibiting apoptosis in vitro. In vivo studies proved that POLE2 was positively correlated with ESCC tumor formation, which was consistent with the results in vitro.

We also illuminated that POLE2 knockdown upregulated pro-apoptotic proteins and downregulated anti-apoptotic proteins, which paved a path to the regulatory mechanism of cell apoptosis by POLE2. It was well known that the mechanisms of apoptosis and their effector proteins included pro-apoptotic protein, anti-apoptotic members, and inhibitor of apoptosis proteins [19]. Bax protein is a member of the multi-domain Bcl-2 family and is directly involved in the initiation of mitochondrial-induced apoptosis [20]. The activation of mitochondrial-mediated apoptosis pathway is the main anti-tumor response of p53 [20]. The p21 can be induced by p53-dependent and p53-independent mechanisms, and its important functions include regulating apoptosis [21]. In addition, Caspase3 is identified as the downstream of p53 [22]. Furthermore, CD40-CD40L interaction can affect the growth regulation of some tumors. Matsumura et al., proposed that the expression of CD40 is associated with progression of ESCC [23]. Moreover, as one of the apoptosis-related markers, FasL plays an important role in the development of ESCC [24]. IGFBP5 is a member of IGF system and a pro-apoptotic factor [25]. The expression of IGFBP5 can reverse cisplatin resistance of ESCC [26]. On the other hand, CLAP-2, IGF-I and sTNF-R2 all anti-apoptotic proteins [27, 28]. IGF-I can act on different apoptosis control points, including Bcl-2 family proteins, caspase inhibitors and death-induced receptor signal transduction [29]. Consistently, knockdown of POLE2 promoted cell apoptosis through upregulating pro-apoptotic proteins Bax, Caspase3, CD40L, FasL, IGFBP-5 and P21, as well as downregulating anti-apoptotic proteins CLAP-2, IGF-I and sTNF-R2 was determined in this study. Accordingly, POLE2 was participated in apoptosis induction of ESCC requiring the regulation of varieties apoptosis-associated factors.

Furthermore, we proved that knockdown of POLE2 is associated with the expression of AKT, p-AKT, Cyclin D1, PIK3CA and MAPK9 in ESCC. In the analysis of molecular mechanism, Xu et al. revealed that TEX9 and eIF3b promoted the progression of ESCC through the activation of AKT signaling pathway [30]. Li et al. has been previously reported the importance of the PI3K/AKT signaling pathway in ESCC metastasis and support PI3K/AKT as a valid therapeutic target in treatment of metastatic ESCC [31]. Hong et al. proposed that high level of Cyclin D1 expression increased distant metastasis, decreased overall survival and distant metastasis-free survival in ESCC [32]. PIK3CA mutation occurs frequently in patients with ESCC, which is associated with poor prognosis [33]. MAPK9 is a tumor suppressor in oral cancer, emphasizing its positive role in apoptosis [34]. Additionally, we found that the downregulation of POLE2 resulted in the decreased expression of Akt, p-Akt, Cyclin D1 and PIK3CA, and the increased expression of MAPK9 in ESCC.

## Conclusions

Although we have provided valid evidence indicating the role of POLE2 in ESCC, this study is still limited. For example, the number of clinical specimens included in this study is small and the underlying mechanism of POLE2 mediated regulation of ESCC is still not clear. Therefore, we will further deepen the understanding of POLE2 related molecular mechanism of ESCC in the future work.

In a nutshell, we elucidated that POLE2 may be associated with the development and prognosis of ESCC. Therefore, this study suggested that POLE2 may be a potential therapeutic target, providing a new therapeutic strategy for preventing or delaying the progression of ESCC.

## Data Availability

All data generated or analysed during this study are included in this published article.
